# Substitutional Analysis of the C-Terminal Domain of AbrB Revealed Its Essential Role in DNA-Binding Activity

**DOI:** 10.1371/journal.pone.0097254

**Published:** 2014-05-15

**Authors:** Svetlana Neubauer, Olga Dolgova, Gregory Präg, Rainer Borriss, Oliwia Makarewicz

**Affiliations:** 1 Institute of Biology, Humboldt University of Berlin, Berlin, Germany; 2 Center for Infectious Diseases and Infection Control, Jena University Hospital, Jena, Germany; University of Groningen, Groningen Institute for Biomolecular Sciences and Biotechnology, Netherlands

## Abstract

The global transition state regulator AbrB controls more than 100 genes of the *Bacillus* relatives and is known to interact with varying DNA-sequences. The DNA-binding domain of the AbrB-like proteins was proposed to be located exclusively within the amino-terminal ends. However, the recognition of DNA, and specificity of the binding mechanism, remains elusive still in view of highly differing recognition sites. Here we present a substitutional analysis to examine the role of the carboxy-terminal domain of AbrB from *Bacillus subtilis* and *Bacillus amyloliquefaciens*. Our results demonstrate that the carboxy-terminal domains of AbrB affect the DNA-binding properties of the tetrameric AbrB. Most likely, the C-termini are responsible for the cooperative character observed for AbrB interaction with some DNA targets like *tycA* and *phyC*.

## Introduction

The transition from exponential bacterial growth into the stationary phase requires many rearrangements of gene expression to ensure survival under growth-limiting conditions. AbrB is the best studied key transition state regulator of the *Bacillus* species and it is known to regulate more than 100 post-exponentially expressed genes, encoding for different cell functions like sporulation [1], biofilm formation [2], antibiotic production [3] or development of competence [4]. This tetrameric protein acts mainly as a repressor, only few genes are known to be activated by AbrB [5], [6], [7]. However, since no satisfying recognition site could be evaluated the mechanism of the interaction of AbrB with its targets is still unclear [8]. It was hypothesized that AbrB-like proteins may recognize specific three-dimensional DNA helix configurations (like minor groove width or degree of propeller twisting) rather than a specific base sequence [9], [10].

AbrB binds to its targets with different affinities [10], [11] and different cooperativities [9], [11], [12], [13], [14]. The recognized DNA sequences show a high AT-content, but an intrinsic bend is not prerequisite for AbrB binding [15]. The high AT-content might support the inherent flexibility of the DNA to alter its conformation [16] while bound to AbrB [8], [10].

Although, AbrB exhibits promiscuous DNA binding properties it also shows high specificity [9], [10], [12], [17], [18]. An *in vivo* genome wide profile analysis revealed that AbrB and its paralogous Abh recognized hundreds of sites as homomer and/or heteromer forms with different specificities throughout the entire genome. But only around 42% of the binding sites are located within the non-coding regions [19]. Moreover, from 643 AbrB-binding sites, only 103 have been suggested to directly affect transcription. This raised the controversial idea that AbrB-like transcription factors might primarily act as nucleoid-associated proteins, similar to H-NS and its paralog StpA described in *Escherichia coli* [20], [21].

The active AbrB protein is a tetramer of identical subunits [10], [22], [23]. The N-termini (aa 1 to 53) of two monomers form dimers that in turn interact via the free C-termini (aa 54–94) to form tetramers [23], [24]. The N-terminal domain can easily be expressed in *E. coli* cells, and can be purified as a stable dimer AbrBN that binds specifically to the target DNA *in vitro*. The NMR solution structure of the N-terminal dimer has already been solved, revealing a ‘swapped hairpin β-barrel’ DNA-binding motive related to double-ψ-β-barrels of VatN-N [25], [26]. Based on the mutational model of the MazE-DNA-complex [27] the DNA-binding site of AbrBN was proposed to consist of the conserved arginine residues (R8, R15, R23 and R24) located around the β2/β2' pair [25]. Some experimental substitution data of the N-terminal domain supported the DNA-binding functions of selected amino acid residues [23]. While the N-terminal domain of AbrB is well studied the overall structure of the tetramer seems to be more challenging, most probably due to the independent character of both domains [23], [28]. However, recently Olson and co-workers presented a NMR-structure of the C-terminal domains [28]. By using LC/MS^n^ data of cross-linked trypsin-digested fragments of AbrB they could predict the position of the N-terminal dimers in relation to the C-terminal dimers thereby modeling a possible full-length structure of the AbrB tetramer. The predicted AbrB-tetramer favors the hypothetical structure [29], [30], but both domains have been individually solved thus the accurate conformation of the tetramer remains uncertain.

Currently, it is accepted that the C-terminal domains of AbrB and its paralogue Abh possess only tetramerization functions, thereby stabilizing the active tetrameric form of the proteins. This idea is supported by experiments with chimeric proteins of the N-terminal AbrB-domain fused to the C-terminal Abh or SpoVT domains that in contrast to the truncated AbrBN were still able to repress the *abrB*8-promoter *in vivo* [18], [23]. Substitutional and deletion mutant proteins indicated that some amino acid residues (C54, N64 and L67), as well as the last residues (aa 81 to 94) of the C-terminus are essential for tetramerization and consequently also for proper protein function [18], [22], [23].

**Figure 1:**
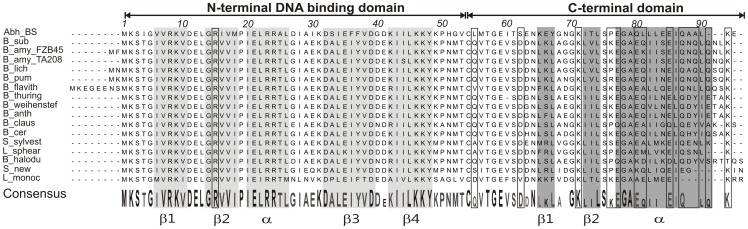
Amino acid alignment of AbrB-like proteins of *Bacillus* related species. N- and C-terminal domains are indicated on the top. Known conserved structures of the DNA-binding domain (β1-4 and α) are highlighted in gray according to (7). The structured regions (β1-2 and α) of the C-terminal domain of AbrB from *B. subtilis* are highlighted in dark gray according to Olson *et al.* [28]. Amino acid residues that were subjected to the substitution are framed. The consensus sequence resulting from this alignment is given on the bottom and the sizes of the amino acid code indicate the percentage of the similarity (100%, 77%, 65% and 42%). Abh_BS  =  Abh protein of *B. subtilis* 168, B_sub  =  *B. subtilis* 168, B_amy_FZB45  =  *B. amyloliquefaciens* FZB45, B_amy_TA208 =  *B. amyloliquefaciens* TA208, B_lich  =  *B. licheniformis* ATCC14580, B_pum  =  *B. pumilus* SARF-032, A_flavith  =  *Anoxybacillus flavithermus* WK1, B_thuring  =  *B. thuringensis* Al Hakam, B_weihenstef  =  *B. weihenstefanansis* KBAB4, B_anth  =  *B. anthracis* A0248, B_claus  =  *B. clausii* KSM-K16, B_cer  =  *B. cereus* ATCC14579, S_silvest  =  *Solibacillus silvestris* StLB046, L_shearic  =  *Lysinibacillus sphearicus* C3-41, B_halodu  =  *B. halodurans* C-125, S_new  =  *Sporosarcina newyorkensis* 2681, L_monoc  =  *Listeria monocytogenes* L312

Among the rising number of identified AbrB-like proteins, only Abh and SpoVT have been thoroughly analyzed. Interestingly, AbrB and SpoVT specifically discriminate between their own DNA-binding sites, albeit they share 80% similarity in the N-terminus. There are only a small number of non-conserved amino acid residues between residues 1 and 53 of both proteins, but the C-terminal domains are not related. The N-terminal domains of Abh and AbrB are highly conserved (Fig. 1) bearing approximately 94% similarity (with 70.4% of identical residues). On the other hand the C-termini of AbrB and Abh share less similarity (34% identical residues). Both proteins as well as their heterodimers share overlapping binding sites, but they also exhibit significant preferences to specific motifs [19]. Thus, beside their multimerization functions the C-termini of AbrB-like proteins could influence DNA-binding activity, not least due to the essential role of the tetrameric state in the DNA-binding activity of swapped-hairpin transcription factors [10], [31].

To analyze the impact of some residues of the C-terminal domain of AbrB on the polymerization's state and binding activity we performed an alanine screening mutagenesis. For the binding assays we chose two different targets due to the different nature of their AbrB binding sites. We used the *phyC* promoter region which contains two distant, cooperative AbrB binding sites, ABS1 and ABS2 (440 bp) [12], [14] as well as the known AbrB-binding site (36bp) of *sinIR* [32]. We also analyzed some substitutions to amino acids with an opposite charge. Our results presented here support the idea of a functional impact of the C-terminal domains in the AbrB-DNA-interactions.

## Material and Methods

### Bacterial strains, plasmids, and media

Bacterial strains and plasmids used in this study are listed in Table 1. Strains were grown in a Lysogeny Broth (LB) medium, when appropriate ampicillin (Ap) was added to a final concentration of 100 mg/L.

**Table 1:** Strains and plasmids used in this study

*E. coli* strains	Description	Reference or source
XL gold	*tetRΔ(mcrA)183 Δ(mcrCB-hsdSMR-mrr)173 endA1 supE44 thi-1 recA1 gyrA96 relA1 lac Hte [F' proAB lacIqZΔM15 Tn10 (TetR) Amy CamR]*	Agilent Technologies
BL21 (DE3)	F *ompThsdS* (*r m* ) *gal dcm* (DE3)	Novagen
ECAbrBBS	F_ *ompT hsdSB*(rB mB ) *gal dcm* (DE3)::pAbrB	[12]
ECAbrBBA	F_ *ompT hsdSB*(rB mB ) *gal dcm* (DE3)::pAbrBQ81K	[12]
*Plasmids*		
pABRB	pET15b with *abrB* (AS 1-94) of *B. subtilis*, fused to His_6_-tag, Substitutions of pABRB: All mutagenized plasmids were named according to the introduced substitution e.g.: pR15A means arginine at position 15 was exchanged by alanine.	[12] and this work

### DNA manipulations and general methods

PCR-products were purified using the NucleoSpin-Extract II kit (Machery Nagel) and plasmids were isolated using the NucleoSpin-Plasmid kit (Machery Nagel). Endonuclease digestions and PCRs were conducted using enzymes purchased from Fermentas, according to manufacturer's instructions. Agarose gel electrophoresis was performed in 1xTAE (40 mM Tris, 1.1 ml/l acidic acid, 1 mM EDTA, 0.5 µg/ml ethidium bromide) buffer.

Radioactive gels were vacuum dried at 80°C, exposed on a phosphor-screen (Kodak), and visualized on a Molecular Imager FX-Pro Plus (BioRad).

### Site directed mutagenesis

Plasmids carrying substitutions within *abrB* (for details see table 1) were generated using the QuikChange XL Site-Directed Mutagenesis Kit (Agilent Technologies) and pABRB [12] as template according to manufacturer's instructions. The mutations were confirmed by sequencing of the *abrB* insert using the standard sequencing T7-promoter and T7-terminator primers (SMB, Berlin, Germany). Ca^2+^ competent *E. coli* BL21 cells ware transformed with appropriate plasmids according to Sambrook *et al.* [33]

### Over-expression and purification of the AbrB proteins

*E. coli* BL21 cells bearing the expression plasmids were grown in LB, by shaking at 200 rpm and at 37°C up to an OD_600_ of 0.8. By adding 1 mM isopropyl β-D-1-thiogalactopyranoside (IPTG) into the cultures the His_6_-AbrB proteins were expressed for a further 3 hours under the same conditions. The cells were harvested in a Sorvall RC5B centrifuge (Thermo Scientific) at 5000 rpm (in a GS rotor) and washed with 100 ml H-buffer (50 mM HEPES pH 7.5, 300 mM NaCl, 2 mM β-mercaptoethanol, 10% glycerol). The cell pellet was resuspended in 10 ml H-buffer and incubated with 1 mM phenylmethylsulfonyl fluoride (PMSF) (Serva) and 25 U benzonase (Novagen) on ice for 20 min. Lysis of cells was carried out by sonication. Purification of His_6_-AbrB variants was performed by Ni^2+^-NTA (Qiagen) followed by Protino Ni-TED 1000 (Macherey-Nagel) chromatography. After each purification step the proteins were dialyzed against 50 mM HEPES (pH 7.5), 300 mM NaCl, 2 mM β-mercaptoethanol, and 10% glycerol. The quality of the proteins was analyzed in 14% SDS-polyacrylamide electrophoresis and pure fractions were pooled and dialyzed against 50 mM HEPES (pH 7.5), 300 mM NaCl, 2 mM β-mercaptoethanol, and 50% glycerol. Protein concentrations were measured at 280 nm in a photometer (NanoDrop ND2000, Peqlab Biotechnologie GmbH) and conversed using the predicted extinction coefficient of 2560/(M cm). Additionally, tyrosine fluorescence was used to adjust the concentrations of all proteins for gel shift experiments.

### Fast protein liquid chromatography (FPLC)

The polymerization states of the AbrB-variants were analyzed by analytical gelfiltration on a Superdex 75 5/150 GL column with a bed volume of 3 ml (ÄKTA, GE Healthcare). All proteins were analysed in running buffer condition I (50 mM HEPES pH 7.5, 300 mM NaCl, 2 mM β-mercaptoethanol) by applying constant volumes of 100 µl proteins. For proteins that tend to aggregate (R15A, K71A, E77A, E80A, Q91A, Q55E, Q81E and K94I) the FPLC was repeated in running buffer supplemented with 10% glycerol (condition II) and 60 µl of the proteins (corresponding to 100 µg) were applied. The wild-type AbrB was analyzed under both conditions. The analytical column was run with a flow rate of 0.2 ml/min. The protein content of the flow through was detected at 280 nm. The calibration was performed with a mixture of standard proteins with known molecular masses: vitamin B12 (1.35 kDa), myoglobin (17 KDa), ovalbumin (44 kDa), and alpha-globulin (158 kDa). The quantification of the polymeric AbrB-forms were calculated as the areas under the curve (AUC) and expressed as the ratio (in %) to all protein peaks per chromatogram (100%).

### Double stranded oligonucleotide *sinIR*

A 36 bp DNA fragment (*sinIR* AbrB binding site) corresponding to the *sinIR* P_1_ promoter region +20 to +55 was prepared using single stranded complementary oligonucleotides SinIRAbrBfor (GTGATTTAATGGCAAATGACTTCCAGAGACTAATGA) and SinIRAbrBrev (TCATTAGTCTCTGGAAGTCATTTGCCATTAAATCAC) [32] (obtained from Eurofins MWG Operon, Germany). The complementary strands were dissolved in water to obtain 100 µM stock solutions and mixed 1∶1. Double-stranded DNA was produced by three denaturation steps at 96°C for 5 min and chilling to RT. The quality of the ds-oligonucleotides was analyzed on a 10% TBE-acryl amide gel.

### Gel shift assay

A 440 bp DNA fragment corresponding to the *phyC* region (−333 to +107) was amplified using the primers Sn5 (TCGTGAAAAAA CGGTTGTAGC) and Sn6 (TTGCCC TGGGAAGAAACC) and the *Pwo*-Polymerase (Peqlab) according to the manufacture's protocol. This DNA fragment and the *sinIR* ds-oligonucleotide were 5′-^32^P labelled by [γ-^32^P]-ATP (Hartmann Analytic) using T4 polynucleotide kinase (Fermentas) and purified using the QIAquick PCR Purification Kit (Qiagen) according to manufacture's protocols, respectively. The binding reaction was carried out in binding buffer (20 mM TrisHCl buffer (pH 8), 100 mM KCl, 5 mM MgCl_2_, 1 mM dithiothreitol, 10% glycerol, and 0.1 mg/ml poly(dI-dC) as a competitive non-specific DNA) with 10000 cpm DNA (corresponding to 1.14 nM of *phyC* or 0.95 nM of *sinIR*), and various AbrB concentrations (0.5 µM–6 µM for *phyC* and 0.01 µM–4 µM for *sinIR,* diluted in H-buffer) for 20 minutes at room temperature. The reaction mixtures were separated on 6% non-denaturing polyacrylamide gels in 1 x Tris-borate-EDTA buffer at 100 V, dried, and exposed on a phosphor screen. Images were visualized and quantified using a Molecular Imager FX pro plus (BioRad) and the Quantity One software (BioRad). If required, optical densities of free and AbrB-bound DNA were determined and binding affinities, expressed as apparent equilibrium dissociation constant K_D’_, were calculated based on plotting Lg [(DNA_bound_)/(DNA_free_)] versus Lg [AbrB_4_] as the interception with the X-axis.

## Results

### Selection of the amino acid residues to be substituted

According to the work of Luscombe *et al.*, particular amino acids interact preferentially with specific bases [34]. They could show that mainly arginine (R) and lysine (K) and to a lower extent glutamine (Q) and asparagine (N) are involved in interactions with DNA-bases, as well as with DNA-backbone by forming multiple bonds between an amino acid side chain and one or more bases [34]. Aspartic acid (D) and glutamic acid (E) can also interact with the DNA-bases in a complex manner. Thus, we focused on R, K, Q, N, D and E as the preferred amino acid residues in our study. The multi-sequence alignment (ClustalW) of AbrB from various *Bacillus* species and some relatives (see Fig. 1) including the AbrB-paralogous protein Abh of *B. subtilis* revealed highly conserved N-termini, whereas the C-termini exhibited more variability. Conserved amino acids that exhibit either polar (Q55, Q87) or charged residues (D62, K71, E77, E80, E85, and K94) as well as the less conserved residues N88, K76, Q81, Q89 and Q91 were chosen for alanine exchange within the C-terminal domain. Generally, we did not exchange the strongly conserved residues E59 and N64 or small and hydrophobic amino acids due to their ‘backbone’ functions which could interfere with the integrity of the protein. D63 and K66 were not substituted since both were less conserved. Conserved residues K71 and K94 were of particular interest, since all AbrB-like proteins bear K71 and most proteins bear lysines (one or more) at the C-terminal ends. In total, 14 alanine substituted mutants of AbrB were constructed. We also substituted the highly conserved N-terminal arginine (R15A), which is proposed to be involved in DNA interactions [29]. Furthermore, we exchanged two polar glutamines with the negatively charged (acidic) glutamic acids (Q55E and Q81E) as well as a polar asparagine with the negatively charged aspartic acid (N88D). We also included the naturally-occurring substitution Q81K of *B. amyloliquefaciens*. Finally, the conserved terminal basic lysine was replaced by the hydrophobic isoleucine (K94I).

### Polymerization state of the substituted AbrB proteins

We used an analytical, high-resolution gel filtration (FPLC, for details see Methods) to determine the polymerization states of the wild-type AbrB and of the substituted proteins to quantify the occurring polymeric forms. The percentage of the respective polymeric state was calculated from the areas under the curve (AUC) of each chromatogram (Table 2, and Figures S1 and S2). The wild-type AbrB showed 87% of tetrameric protein and 13% of higher-order octamers without glycerol in the running buffer (condition I) and >95% of tetramers when 10% glycerol was added (condition II). Interestingly, most of the alanine substitutions within the C-terminus did not cause any significant changes in the polymeric state of the proteins compared to the wild-type. The mutants exhibited comparable ratios of tetrameric to higher order polymers: K71A and Q91A with 85–87%/>95% (− glycerol/+ glycerol) tetramers, Q55A, D62A, Q81A, E85A, Q87A, N88A, Q89A, Q91A and K94A with 91–98% tetramers without glycerol. The chromatograms of mutant K76A and naturally-occurring AbrB-variant Q81K suggested nearly 100% of tetramers without glycerol. Mutants E77A and E80A exhibited 17 to 22% of octamers without glycerol or 6% to 10% higher polymers (51–55 subunits) with glycerol indicating aggregation processes. By contrast, an alanine substitution of R15A within the N-terminus caused a strong increase of octameric forms up to 42.5% under glycerol-free buffer condition and elongated plateau covering a broad range of polymeric forms (1 to 12 subunits) and a peak at approximately 40 subunits in the presence of glycerol.

**Table 2:** Relative molecular masses [kDa] and polymerization states (n) of substituted AbrB-variants (for detailed chromatograms see Figure S1 and Figure S2).

buffer condition I				buffer condition I				buffer condition II			
**AbrB**	**kDa**	**n**	**%**	**AbrB**	**kDa**	**n**	**%**	**AbrB**	**kDa**	**n**	**%**
**Q55A**	50±2.99	3.9	92	**wt**	51.2±5.68	4.1	87	**wt**	60.6±6.03	4.8	>95
	102.8±4.99	8.1	8		102.8±4.99	8.2	13				
**D62A**	51.8±3.09	4.1	94	**R15A**	50.9±2.95	4.1	57.5	**R15A**		12< n >1	90
	102.8±4.99	8.2	6		99.1±5.76	8.0	42.5		507±49.3	40.4	10
**K76A**	50.6±3	4.0	>95	**K71A**	53.6±2.6	4.3	86	**K71A**	49.3±5.24	3.9	>95
**Q81A**	55.5±3.31	4.4	94		106.4±5.17	8.5	14				
	106.4±5.17	8.5	6	**E77A**	51.8±3.75	4.1	83	**E77A**	59.2±4.9	4.7	94
**E85A**	50 ± 4.05	4.0	98		102.8±4.99	8.2	17		698.1±55.23	55.5	6
	102.8±4.99	8.2	2	**E80A**	49.2±2.32	3.9	78	**E80A**	56.7±5.54	4.5	90
**Q87A**	56.7±3.38	4.5	93		102.5±4.84	8.2	22		708.3±66.11	56.3	10
	101.5±4.91	8.1	7	**Q91A**	59.4±3.55	4.7	85	**Q91A**	51.6±6,71	4.1	>95
**N88A**	57.1±2.78	4.5	91		102.8±4.99	8.2	15				
	102.3±4.98	8.1	9	**Q55E**	54.2±2.61	4.3	49	**Q55E** c	47.2±3.75	3.8	>95
**Q89A**	61.1±3.65	4.8	93		155.8±7.52	12.3	51				
	102.3±4.98	8.1	7	**Q81E**	49.2±3.37	3.9	78	**Q81E** b	51.6±4.28	4.1	>95
**K94A**	51.2±3.05	4.1	93		102.5±4.84	8.1	22				
	102.8±4.99	8.2	7	**K94I**	59.1±3.65	4.7	65.6	**K94I**	51.7±5.01	4.1	>95
**Q81K** a	50.1±4.86	4.0	>95		171.4±8.3	13.6	34.4				
**N88D**	51.2±3.6	4.1	93								
	105±5.08	8.3	8								

a Q81K corresponds to the wild type of *B. amyloliquefaciens* FZB45.

b Q81E showed a plateau at lower retention volumes containing octamers or larger polymeric forms.

c Q55E showed aggregation under different conditions and was stable only in presence of 5 mM β-mercaptoethanol.

Substitution of the polar, but uncharged N88 by the negatively charged aspartic acid (D) had no effect on the tetramerization (93%), but some octamers (8%) were also found without glycerol. Substitutions of polar, but neutral amino acid residues Q55 and Q81 by negatively charged glutamic acid residues (E) triggered polymerization to larger polymeric forms under glycerol-free conditions. For Q55E more than 50% of higher order polymers (possibly up to 12 monomers) were determined. Q81E showed 78% of tetramers and a plateau at lower retention volumes containing 22% of octamers (or higher-order polymers). In presence of glycerol both proteins showed >95% of tetrameric proteins. Exchange of the positively charged K94, that represents the last residue of the protein, with the non-polar and hydrophobic isoleucine also caused an increase (up to 30%) towards larger polymeric forms (possibly up to 14 monomers) but in presence of glycerol >95% of the protein was tetrameric.

Thus glycerol seemed to stabilize most of the proteins, but had a destabilizing effect of R15A. To sum up, only mutant R15A showed significant differences in its polymeric state.

### Alanine substitutions within the C-terminus of AbrB strongly influenced the binding ability to *phyC*

**Figure 2:**
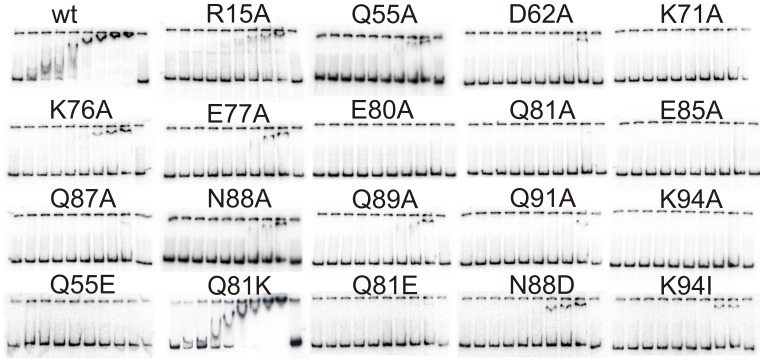
Gel shift assays of various AbrB-mutants bound to the *phyC*-region. The AbrB-protein concentrations (from left to right) for each gel were 0 µM, 0.5 µM, 0.75 µM, 1 µM, 1.5 µM, 2 µM, 3 µM 4 µM, 6 µM, and 0 µM. The substitutions are specified above each gel, wt indicates the native AbrB-protein of *B. subtilis* 168 and AbrB BA corresponds to the protein of *B. amyloliquefaciens* FZB45.

Wild-type AbrB of *B. subtilis* as well as the mutant protein AbrBQ81K, identical with the AbrB of *B. amyloliquefaciens* FZB45, shifted the *phyC* DNA at concentrations higher than 0.5 µM and showed smearing bands up to 1.5 µM (Fig. 2) similar to previous gel retardation studies [12], [35]. At concentrations higher than 2 µM a super shift was observed (Fig. 2). This behavior indicates the formation of intermediate complexes at moderate concentrations and the formation of higher complexes when saturated with AbrB. Alanine substitutions of polar or charged residues resulted in similar amounts of tetramers as for the wild-type AbrB, but exhibited strongly reduced or no DNA-binding activity. Mutants substituted at Q55A, K76A, E77A, N88A, and Q89A bound to *phyC* at concentrations higher than 2 or 3 µM. Mutants with exchanges D62A, Q81A, Q87A, and Q91A shifted *phyC* at above 6 µM protein. Alanines at positions K71A, E80A, E85A, and K94A completely abolished the ability of the mutant proteins to bind at *phyC*. The selected AbrB concentrations approximated physiological conditions for signaling proteins that have been described in a range of 10 nM to 1 µM [36]. Under these gel retardation conditions, it was impossible to calculate any binding constants for these mutants. However, the results clearly demonstrated that the C-terminal domain of AbrB is involved, either directly or indirectly, in the interaction with DNA.

### Alanine at position R15 favored higher order AbrB-polymers but did not abolish DNA binding

Substitution of arginine R15 to alanine resulted in an increase of octameric forms of AbrB. However, this mutant protein was still able to shift *phyC* DNA. For this mutant and the wild-type AbrB apparent dissociation constants (K_D’_) were calculated, based on the optical densities of the free and AbrB-bound DNA. Considering that the concentration of the tetrameric protein of AbrBR15A and the wild-type AbrB was only 57.5% and >95% respectively, the binding activity was only five-fold reduced (3.4×10^−6^ M) compared to the wild-type (0.66×10^−6^ M) (plots see Figure S3). These K_D’_ values pinpointed the effect of the R15A substitution on DNA-binding that was expected to be stronger. Based on NMR-structural data, R15 was proposed to be primarily involved in DNA-binding and dimerization of the N-termini [25], [29]. But it seems to have also an impact on tetramerization functions.

### Negatively charged residues at position Q55 and Q81 abolished DNA-binding

Whilst the alanine substitution of the polar glutamine Q55 led to reduced binding properties, an exchange to a negatively charged glutamic acid (Q55E) abolished the binding activity of the mutant protein. Similarly, the substituted mutant protein AbrBQ81E lost the ability to bind to *phyC*. Interestingly, the native AbrB variant Q81K of *B. amyloliquefaciens,* bearing a positively charged lysine residue instead of neutral glutamine, showed similar behavior to the AbrB protein from *B. subtilis* (Fig. 2 ) [12]Thus, a negative residue at position 81 seems to be unfavorable for DNA-binding activity of the tetramers.

### Replacement of N88 by alanine or aspartic acid residues slightly impaired protein properties

A substitution of the neutral N88 by the negatively charged aspartic acid resulted in a slight reduction of the binding activity of the mutant protein. The effect was comparable to the exchange by a small hydrophobic alanine. Therefore, the charge or size of this residue did not play a crucial role for the binding activity of AbrB at the *phyC* promoter.

### All substituted proteins lost the ability to bind at the *sinIR* recognition site

The ds-oligonucleotide bearing the AbrB recognition site of *sinIR* promoter *P_1_* was bound to AbrB at concentrations higher than 0.1 µM without smearing effects and without a visible super shift. We performed gel retardation assays of *sinIR* ds-oligonucleotide only with those mutants that were still able to bind at *phyC,* since it seemed to be certain that the non-active mutant proteins would not bind at *sinIR*. However, even these substituted proteins were unable to shift the *sinIR* fragment (Figure S4).

## Discussion

Previous studies indicated that the DNA recognition and binding specificity determinants of AbrB are located primarily, or solely, within the N-terminal domains. But it is also known that C-terminal domains are important for the proper function of the protein *in vivo* [10], [18], [37]. To confirm this hypothesis we investigated their role in DNA-binding by substitution of relatively conserved amino acids within the C-terminus of AbrB. In summary we introduced 14 substitutions to alanine, two to glutamic acid, one to aspartic acid, and one to isoleucine (for details see Table 2). The mutated protein variants were compared with the wild-type AbrB proteins of *B. subtilis* and *B. amyloliquefaciens* (Q81K) regarding their polymerization states and their ability to bind at two different targets: the *phyC* promoter (440 bp), and the AbrB site of the *sinIR* promoter P_1_ (36 bp). Previous experiments clearly demonstrated that only tetrameric AbrB is functionally significant [10] and does not change the stoichiomety when bound to DNA [24]. In the present study, the introduced alanine substitutions of the C-terminus did not significantly affect the AbrB tetramerization. By contrast substitution R15A showed shifts to higher polymeric forms, indicating a possible role of this arginine during the tetramerization process. Moreover, substitution R15A retained the binding activity of the protein to the *phyC* promoter. Thus, arginine residue R15 alters the binding affinity but is not crucial for AbrB´s ability to bind DNA [25], [29].

The substituted AbrB proteins showed different binding properties when compared to native AbrB proteins of *B. subtilis* and *B. amyloliquefaciens.* Some substitutions caused reduced binding activities, others abolished the ability to bind the *phyC* promoter. AbrB mutants that still bound to *phyC* showed a poor formation of intermediate complexes that were usually observed for AbrB in DNA-binding assays and indicate a multistep cooperative mechanism [11], [12], [29], [35], [37]. These mutants may be altered in their cooperative character.

Substituted AbrB proteins that exhibited reduced binding activities towards *phyC* but lost the ability to bind at the *sinIR* site, suggest variable interactions of AbrB with different targets. A possible reason might be the size and/or the number of the AbrB-binding sites of the targets (for detailed overview of characterized AbrB-binding sites see Figure S5). AbrB-binding sites longer than 40 bp yielding Hill-coefficients >2 (positive cooperativity) in various binding assays [11], [29], [38] suggesting simultaneous interactions of more than two AbrB tetramers at one DNA-target. The *phyC* gene of *B. amyloliquefaciens*] [12], [14] and the *tycA* gene of *B. subtilis* [13], [39] both are cooperatively repressed by AbrB via two distant binding sites, supporting the idea of complex interaction that involve many AbrB tetramers. This indicates that cooperativity of AbrB might compensate for the DNA-binding defect of some of the substitutions. On the other hand, AbrB interaction with the *sinIR* promoter P_1_ seems to be more elementary since there was no evidence for intermediate complex formation of the unique recognition site spanning only 24 bp [32]. For this site, a 1∶1 (DNA:AbrB tetramer) binding model was proposed, based on PsC-SEC-µESI-MS experiments [30]. AbrB and its truncated N-terminal domain (AbrBN) bound to the *sinIR* recognition site with relatively high affinity, compared to other small native recognition sites (*spo0E* and aptamers) [10]. AbrBN does not exhibit any cooperativity [14] which therefore seems to be mediated through the C-terminal domains. Our interpretation is that binding at short (24bp) sequences like *sinIR* does not necessarily require cooperative interactions [11] but rather a proper conformation of the N-terminal DNA-binding domains, which are dependent on the C-terminal residues. The introduced substitutions might alter the N-terminal conformation. This is in accordance with the previously suggested hypothesis that C-terminal domains trigger the rearrangements within the AbrB-tetramer and stabilize higher-order multimerization [37]. The DNA binding stoichiometries of complexes with long DNA targets like *phyC* or *tycA* have not been determined as yet, neither for AbrBN nor AbrB, but the extensive nature of the AbrB-protected sites in DNaseI footprinting cannot be explained by one tetramer [3].

Comparable to our results, other C-terminal mutants (C54Y, L67P and Q81ter) were defective in DNA binding, thus direct interaction of the C-terminus with DNA was suggested [18]. According to the work of Luscombe *et al.* protein-DNA interactions follow universal rules [34]. Summarized, mainly arginine and lysine, as well as asparagine and glutamine and to a lower content aspartic and glutamic acid residues form hydrogen bonds, van der Waals and water-mediated contacts to the bases as well as to the sugar-phosphate backbone of the DNA. Primary interactions occur between guanines and arginine. Thus, not surprisingly, four highly conserved arginines and seven lysines are present within the N-terminal DNA-binding domain of AbrB. The arginines were already proposed to be crucial for DNA-interactions [25], [29] and AbrB-binding was strongly affected when guanines were chemically modified [14]. There are few non-conserved arginines present within the C-termini of related AbrB proteins, but there is one conserved lysine (K71), one glutamic acid (E59) and one asparagine (N64), as well as some relatively well conserved residues such as glutamines (Q55, Q87), glutamic acids (E77, E80, E85), one lysine K94 and one aspartic acid D62. Formation of hydrogen bonds between amino acid residues and the sugar phosphate backbone is independent of the DNA sequence but stabilizes the protein-DNA complex and may have a role for indirect read-outs by recognizing variations in DNA structure [34]. In our study, mutations K76A, E77A, Q81A, N88A, Q87A, Q89A, and Q91A significantly reduced the binding affinity to *phyC*, but mutations K71A, E80A, E85A and K94A completely abolished the binding activity. Four residues with possible DNA-interaction properties were not substituted in this work (E59, D63, N64, K66). Thus we can not rule out that some polar amino acid residues of the C-terminus might also be directly involved in interaction with the DNA.

However, considering the recent structural data of the C-terminal AbrB domain [28], it seems unlikely that D62, K71, K76, E77, E80, E85 and K94 are involved in DNA interactions. The aspartate residue (D62) is located in the linker region and was proposed to be involved in the dimerization of the C-termini [23]. Residues E77, E80 and E85 are located within the antiparallel α-helices of the domain-swapped dimeric AbrBC. Thus, we hypothesize that these glutamates are involved in charge-packing interactions with the lysine residues of the antiparallel C-terminus (K66 of β1, K71 of loop 1, K76 of loop 2) stabilizing a proper spatial configuration. This could explain the increased octameric forms of mutants E77A and E80A and the partially restored binding activity of mutant K94I in contrast to the non-active K94A. This elongated isoleucine residue might restore intermolecular contacts. The crystal structure of the full-length SpoVT was recently reported, revealing that C-terminal domains are connected to the N-terminal domains by flexible linker sequences, allowing various conformations of the DNA-binding domains [31]. Similar to SpoVT, a linker region connecting AbrBN to AbrBC was proposed for the residues T53–N64 within the N-terminal part of the C-terminus [28]. Thus the reduced or abolished binding activities of the AbrB mutants Q55A/Q55E and D62A might be caused by the reduced flexibility of the tetramer. This idea is supported by other *in silico* studies that suggested a high flexibility in the protein structure, which enables the AbrB/Abh homomers and/or heteromers to adopt various conformations with different specificities and affinities to their targets [19].

*In vivo* investigations of the impact on gene regulation by substituted AbrB-mutants have not extensively been performed so far, due to the challenging nature of this global regulator that influences the key regulatory pathways in *Bacilli*. Only one *abrB* single amino acid substitution that exhibited an *abrB*-negative phenotype could be naturally selected (C56Y) [18]. Analysis of this mutant protein indicated a defective multimerization. Another *in vivo* study described the multimerization potential of the N- and C-terminal AbrB segments, including some substitutions, by using λcI-fusions in an *E. coli* model [23]. Further *in vivo* investigations could help to evaluate which mutations within the C-terminus have a significant impact on the expression profile of the target genes, and how they affect the AbrB/Abh hetero-dimerization. DNA-binding assays and footprinting experiments of the C-terminal dimers would further clarify if the C-terminus specifically interacts with DNA. We did not perform any of these experiments due to expired funding and thus forward the ideas to other scientists working on AbrB.

## Supporting Information

Figure S1 **FPLC analysis of the AbrB variants under running condition I.** Analytical gelfiltration was performed on a Superdex 75 5/150 GL column. Absorbance [mAU] at 280 nm was plotted versus the elution volume [ml]. (A) Run of the wild type protein (violet), calibration run (blue) was performed with a mixture of vitamin B12 (1.35 kDa), myoglobin (17 KDa), ovalbumin (44 kDa), and alpha-globulin (158 kDa). The conversion factor (slope) was determined by the linearized plot (log kDd vs. ml). (B) Calibration runs on various experimental runs. (C) Chromatograms of the AbrB proteis were polled according their protein content. The polymeric size and content of the peaks were calculated with the corresponding calibration/conversion factors.
(TIF)

Figure S2 **FPLC analysis of the AbrB variants under running condition II.** The FPLC was performed similar to Figure S1 except that the running buffer was supplemented by 10% glycerol and the proteins concentrations and volumes were adjusted to 100 µg/60 µl. The calibration runs are indicated for each experiment (A, B, and C). In C two different R15A concentrations were applied (60 µg and 100 µg) without significant changes in the polymeric distribution.
(TIF)

Figure S3 **Determination of the apparent equilibrium dissociation constants (K_D'_) of wild type AbrB and the N-terminal substituted (R15A) mutant.** The optical densities of the free and AbrB-bound *phyC*-DNA were determined and plotted as Lg [(DNA_bound_)/(DNA_free_)] versus Lg [AbrB_4_]. K_D’_ values were determined as the interception with the X-axis.
(TIF)

Figure S4 **Gel shift assays of various AbrB-mutants bound to** ***sinIR*** **-promoter P_1_.** The AbrB-protein concentration (from left to right) for each gel: 0 µM, 0.01 µM, 0.025 µM, 0.05 µM, 0.1 µM, 0.25 µM, 0.5 µM, 1 µM, 4 µM, and 0 µM. The substitutions are specified above each gel, wt indicates the native AbrB-protein of *B. subtilis* 168.
(TIF)

Figure S5 **Overview of the known and characterized AbrB-binding sites.** The corresponding references are specified on the left site and are listed in the main document except: [40] Qian Q, Lee CY, Helmann JD, Strauch MA (2002) AbrB is a regulator of the sigma(W) regulon in Bacillus subtilis. FEMS Microbiol Lett 211: 219–223.; [41] Slack FJ, Mueller JP, Strauch MA, Mathiopoulos C, Sonenshein AL (1991) Transcriptional regulation of a Bacillus subtilis dipeptide transport operon. Mol Microbiol 5: 1915–1925.; [42] Strauch MA (1995) Delineation of AbrB-binding sites on the Bacillus subtilis spo0H, kinB, ftsAZ, and pbpE promoters and use of a derived homology to identify a previously unsuspected binding site in the bsuB1 methylase promote. J Bacteriol 177: 6999–7002.
(TIF)
