# Supplementary figures and images for: Substitutional Analysis of the C-Terminal Domain of AbrB Revealed Its Essential Role in DNA-Binding Activity
Source: PLoS One. 2014 May 15;9(5):e97254. doi: 10.1371/journal.pone.0097254 (PMC4022651; doi:10.1371/journal.pone.0097254)

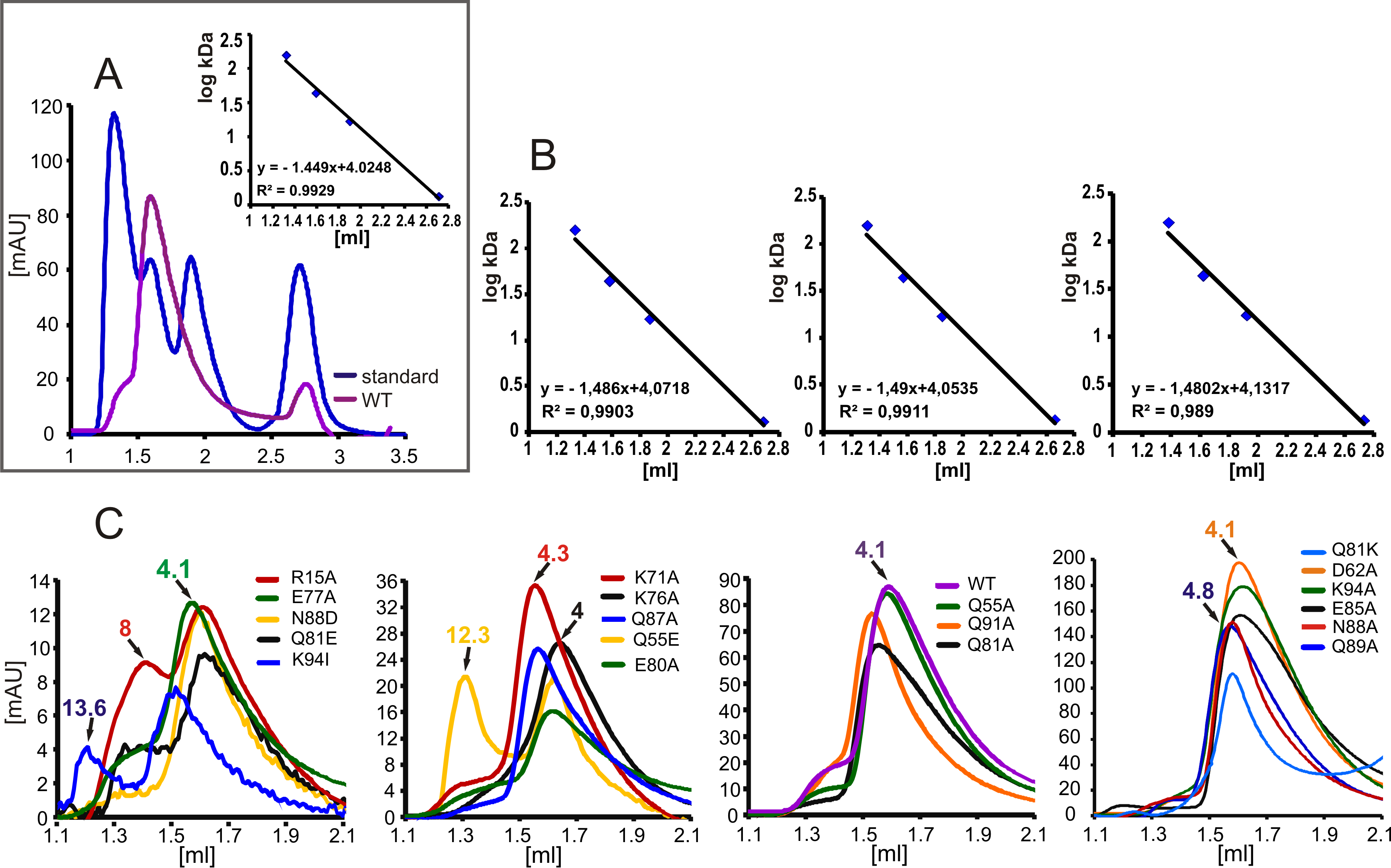

Supplement: Figure S1 — FPLC analysis of the AbrB variants under running condition I. Analytical gelfiltration was performed on a Superdex 75 5/150 GL column. Absorbance [mAU] at 280 nm was plotted versus the elution volume [ml]. (A) Run of the wild type protein (violet), calibration run (blue) was performed with a mixture of vitamin B12 (1.35 kDa), myoglobin (17 KDa), ovalbumin (44 kDa), and alpha-globulin (158 kDa). The conversion factor (slope) was determined by the linearized plot (log kDd vs. ml). (B) Calibration runs on various experimental runs. (C) Chromatograms of the AbrB proteis were polled according their protein content. The polymeric size and content of the peaks were calculated with the corresponding calibration/conversion factors. (TIF) [file pone.0097254.s001.tif]

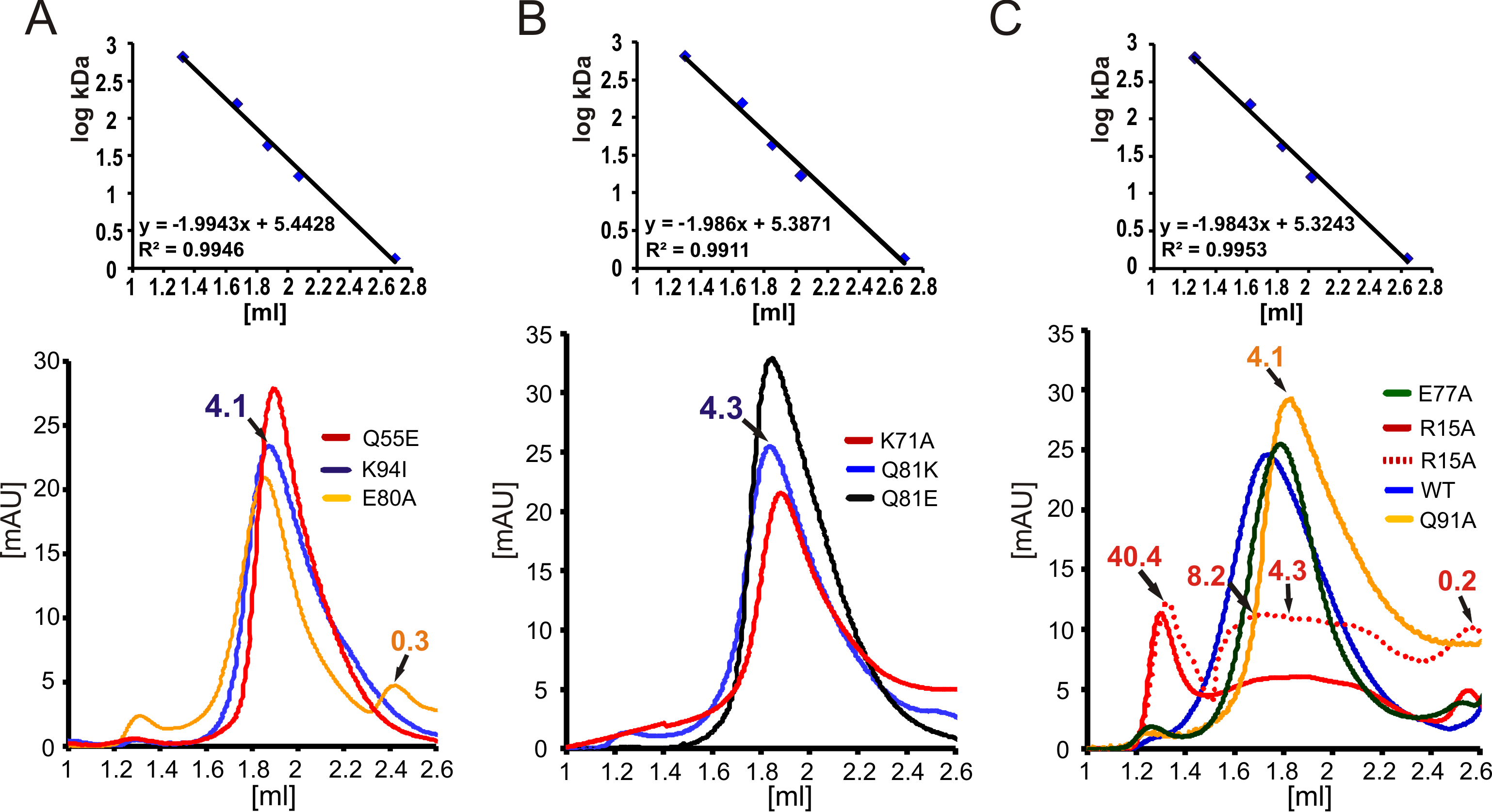

Supplement: Figure S2 — FPLC analysis of the AbrB variants under running condition II. The FPLC was performed similar to Figure S1 except that the running buffer was supplemented by 10% glycerol and the proteins concentrations and volumes were adjusted to 100 µg/60 µl. The calibration runs are indicated for each experiment (A, B, and C). In C two different R15A concentrations were applied (60 µg and 100 µg) without significant changes in the polymeric distribution. (TIF) [file pone.0097254.s002.tif]

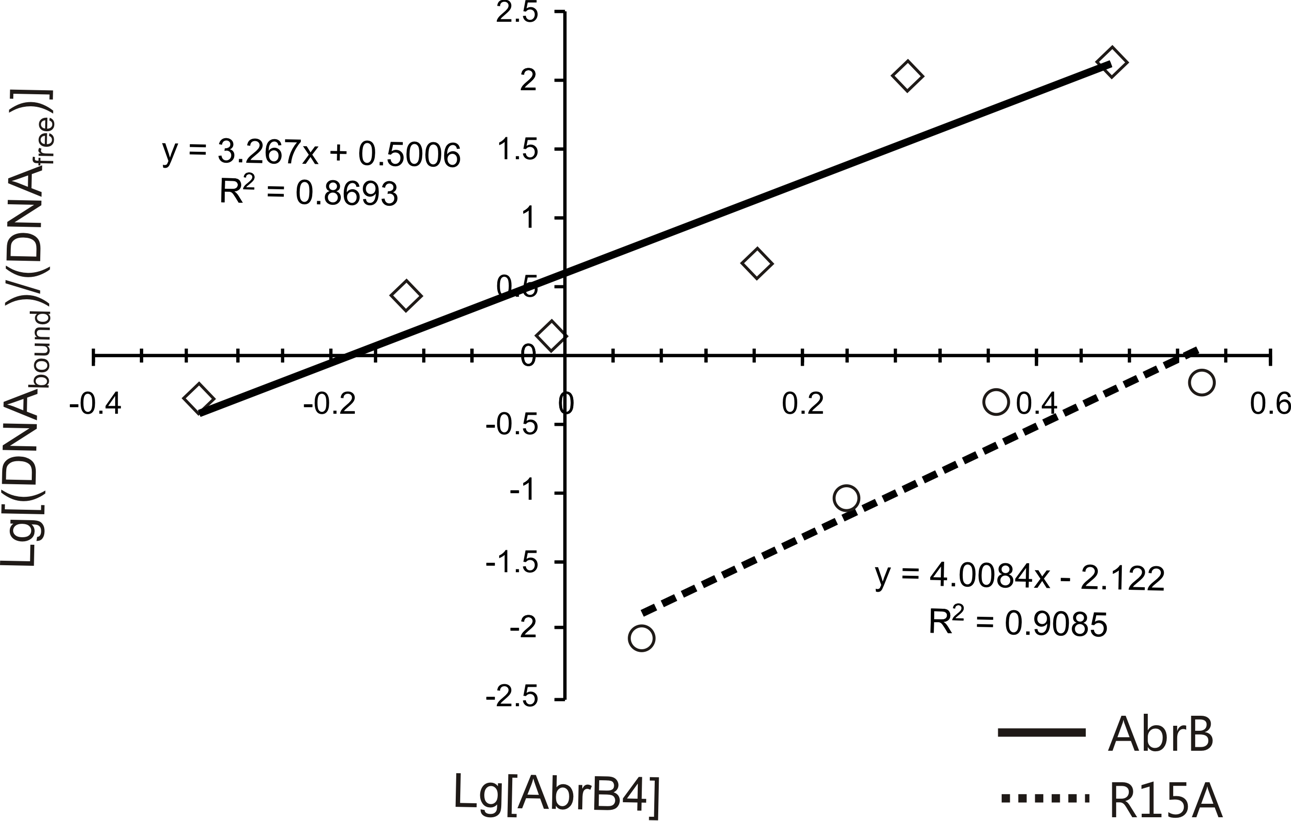

Supplement: Figure S3 — Determination of the apparent equilibrium dissociation constants (KD') of wild type AbrB and the N-terminal substituted (R15A) mutant. The optical densities of the free and AbrB-bound phyC-DNA were determined and plotted as Lg [(DNAbound)/(DNAfree)] versus Lg [AbrB4]. KD’ values were determined as the interception with the X-axis. (TIF) [file pone.0097254.s003.tif]

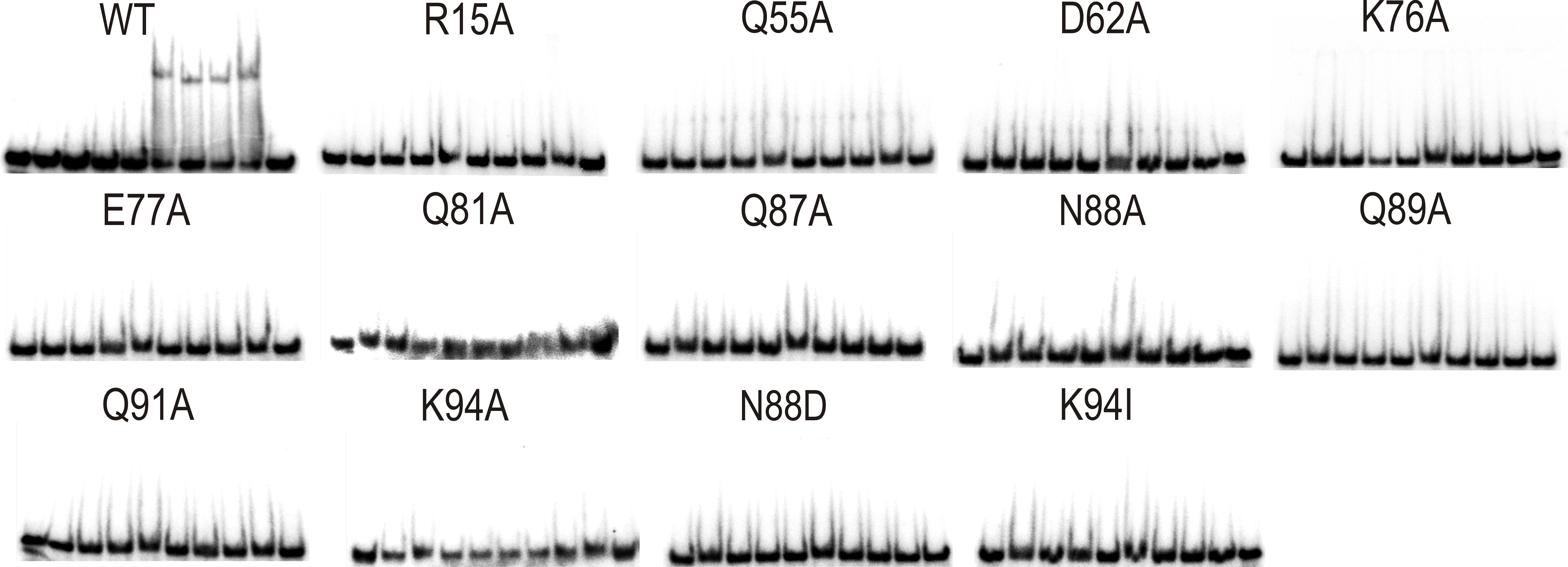

Supplement: Figure S4 — Gel shift assays of various AbrB-mutants bound to sinIR -promoter P1. The AbrB-protein concentration (from left to right) for each gel: 0 µM, 0.01 µM, 0.025 µM, 0.05 µM, 0.1 µM, 0.25 µM, 0.5 µM, 1 µM, 4 µM, and 0 µM. The substitutions are specified above each gel, wt indicates the native AbrB-protein of B. subtilis 168. (TIF) [file pone.0097254.s004.tif]

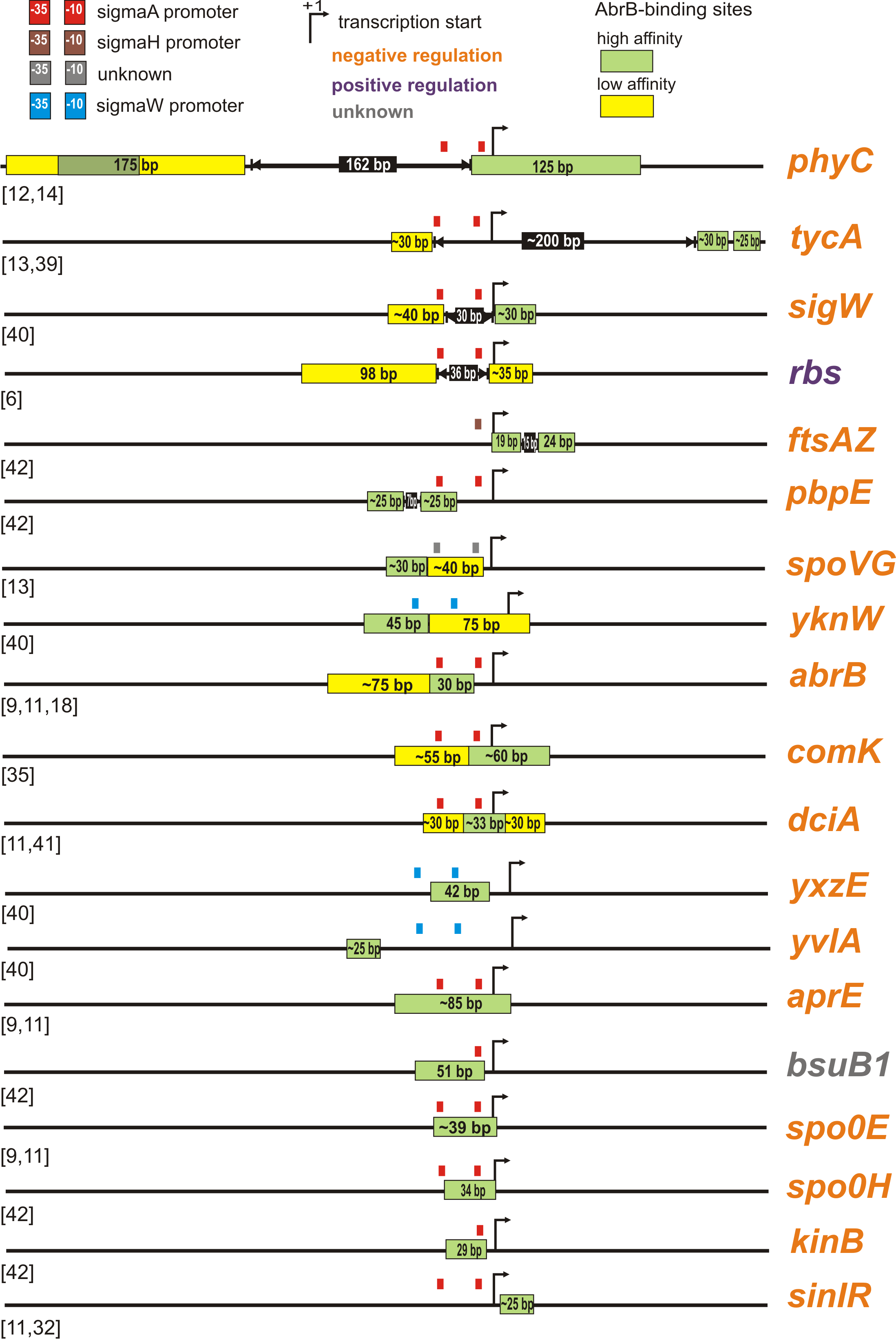

Supplement: Figure S5 — Overview of the known and characterized AbrB-binding sites. The corresponding references are specified on the left site and are listed in the main document except: [40] Qian Q, Lee CY, Helmann JD, Strauch MA (2002) AbrB is a regulator of the sigma(W) regulon in Bacillus subtilis. FEMS Microbiol Lett 211: 219–223.; [41] Slack FJ, Mueller JP, Strauch MA, Mathiopoulos C, Sonenshein AL (1991) Transcriptional regulation of a Bacillus subtilis dipeptide transport operon. Mol Microbiol 5: 1915–1925.; [42] Strauch MA (1995) Delineation of AbrB-binding sites on the Bacillus subtilis spo0H, kinB, ftsAZ, and pbpE promoters and use of a derived homology to identify a previously unsuspected binding site in the bsuB1 methylase promote. J Bacteriol 177: 6999–7002. (TIF) [file pone.0097254.s005.tif]
